# Unraveling the role of C1GALT1 in abnormal glycosylation and colorectal cancer progression

**DOI:** 10.3389/fonc.2024.1389713

**Published:** 2024-04-18

**Authors:** Hong Tian, Jia-Li Yu, Xiaoli Chu, Qi Guan, Juan Liu, Ying Liu

**Affiliations:** ^1^Department of Oncology, Fourth People’s Hospital in Shenyang, China Medical University, Shenyang, China; ^2^Department of Gastroenterology, The First Affiliated Hospital of Dalian Medical University, Dalian Medical University, Dalian, China

**Keywords:** C1GALT1, core 1 β1-3 galactosyltransferase 1, T-synthase, O-glycosylation, colorectal cancer

## Abstract

C1GALT1 plays a pivotal role in colorectal cancer (CRC) development and progression through its involvement in various molecular mechanisms. This enzyme is central to the O-glycosylation process, producing tumor-associated carbohydrate antigens (TACA) like Tn and sTn, which are linked to cancer metastasis and poor prognosis. The interaction between C1GALT1 and core 3 synthase is crucial for the synthesis of core 3 O-glycans, essential for gastrointestinal health and mucosal barrier integrity. Aberrations in this pathway can lead to CRC development. Furthermore, C1GALT1's function is significantly influenced by its molecular chaperone, Cosmc, which is necessary for the proper folding of T-synthase. Dysregulation in this complex interaction contributes to abnormal O-glycan regulation, facilitating cancer progression. Moreover, C1GALT1 affects downstream signaling pathways and cellular behaviors, such as the epithelial-mesenchymal transition (EMT), by modifying O-glycans on key receptors like FGFR2, enhancing cancer cell invasiveness and metastatic potential. Additionally, the enzyme's relationship with MUC1, a mucin protein with abnormal glycosylation in CRC, highlights its role in cancer cell immune evasion and metastasis. Given these insights, targeting C1GALT1 presents a promising therapeutic strategy for CRC, necessitating further research to develop targeted inhibitors or activators. Future efforts should also explore C1GALT1's potential as a biomarker for early diagnosis, prognosis, and treatment response monitoring in CRC, alongside investigating combination therapies to improve patient outcomes.

## 1 Introduction

**Figure 1:**
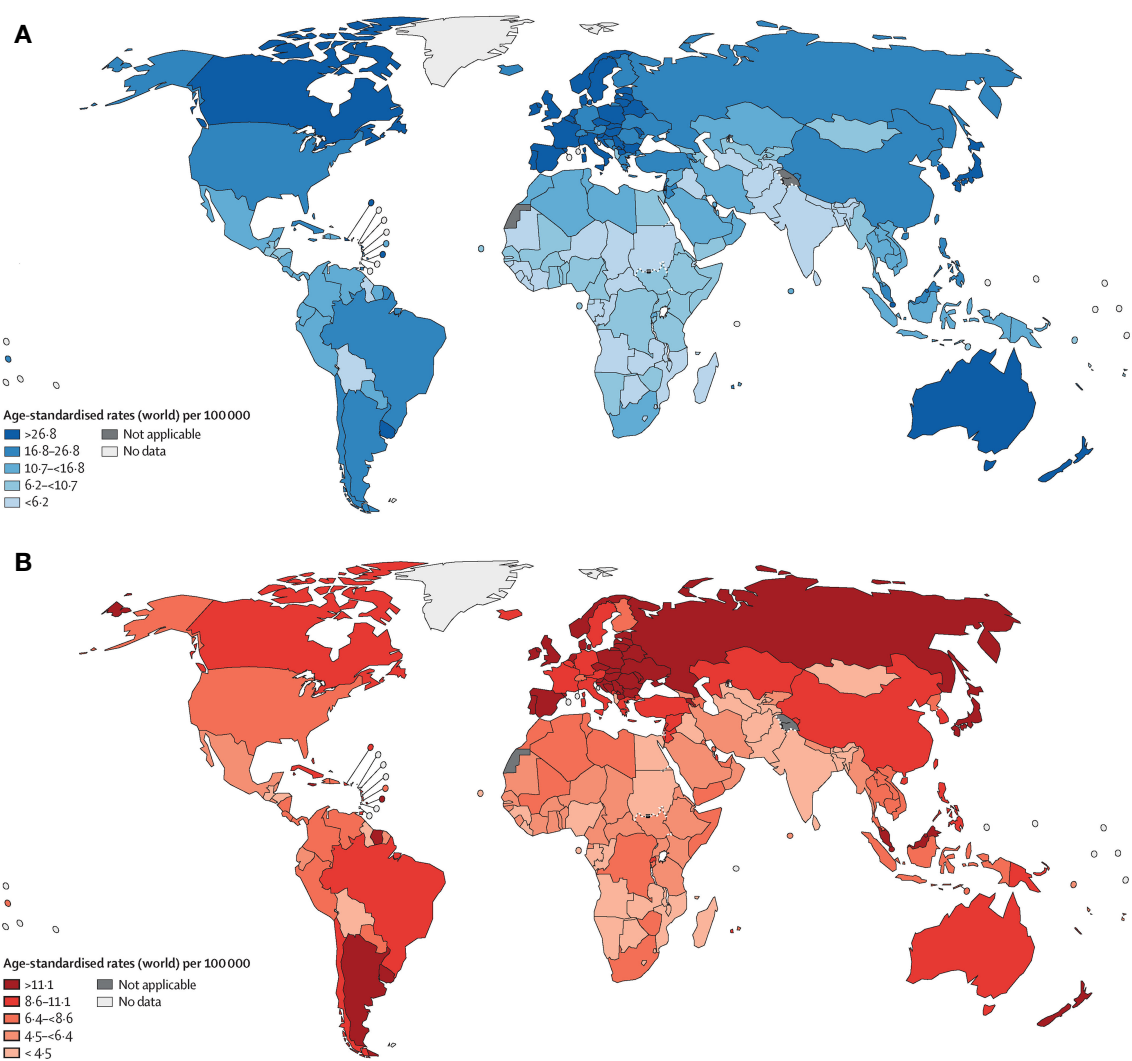
Age-standardised rates of cancer incidence **(A)** and mortality **(B)** across countries in five continents, based on the most recent figures from the WHO International Agency for Research on Cancer (1).

Colorectal cancer (CRC) ranks as the fourth deadliest cancer worldwide, causing nearly 900,000 deaths annually, which accounts for approximately 10% of all annually diagnosed cancers and cancer-related deaths worldwide (1). The incidence and mortality rates vary geographically, with the highest rates seen in the most developed countries (**Figure 1**). Disturbingly, projections indicate a 60% increase in the global burden of CRC by 2030, with an estimated 2.2 million new cases and 1.1 million deaths (2). While advancements in endoscopic technology and screening methods, along with increased early detection efforts, have led to a decline in CRC incidence (3). However, epidemiological studies highlight a worrying trend of increasing colorectal cancer incidence among individuals under 50 years of age (4, 5). Thus, the significant threat posed by colorectal cancer to human health cannot be underestimated. Numerous risk factors contribute to the development of colorectal cancer, including age, male gender, family history, inflammatory bowel disease, smoking, excessive alcohol consumption, high intake of red and processed meat, obesity, and diabetes (6–8). These risk factors often co-occur and interact with each other. Treatment approaches typically involve early surgical intervention for improved prognosis, while stage II and III rectal cancer patients often require surgery-assisted chemotherapy or radiotherapy (7, 9). Notably, surgery combined with targeted chemotherapy has proven effective in treating advanced metastatic disease or post-surgical recurrence (9). Consequently, identifying reliable targets for colorectal cancer represents a promising avenue to enhance survival rates.

Genetic and epigenetic changes are widely recognized as the primary drivers of cancer development, with downstream phenotypic alterations at the protein level playing a crucial role in cancer progression and transmission (10). The alterations includes increased branching of complex and hybrid N-glycans, increased levels of sialyl lewis antigens, truncated O-glycan expression, and complex core fucosylation, which causes anormal expressions of membrane-localized glycans and then leads to malignant transformation in cells (11). Glycosylation, a post-translational modification of proteins, involves the transfer of sugar molecules to proteins through the action of glycosyltransferases, forming glycosidic bonds with amino acid residues (12). It is the most abundant and diverse form of post-translational modification found in all eukaryotic cells (13). Glycopeptide bonds can be categorized into N-linked, O-linked, and C-linked glycosylation, as well as C-mannosylation and generation of GPI-anchored proteins, based on the properties of the linked glycopeptide bonds and oligosaccharides (13). Carbohydrates in the form of N-linked or O-linked oligosaccharides are major structural components of membrane-bound and secreted proteins (14, 15). Through the regulation of protein stability, subcellular localization, activity, and interactions, glycosylation plays a wide-ranging role in key cellular processes, including gene transcription, cell cycle regulation, DNA repair, apoptosis, virus budding, receptor endocytosis, and various physiological and pathological processes (16–18). Tumor cells exhibit extensive glycosylation changes compared to untransformed cells, and glycosylation has been closely associated with various cancers, including hepatocellular cancer (19–21), pancreatic cancer (22, 23), gastric cancer (24), bladder cancer (25), breast cancer (21), esophageal cancer (26), cholangiocarcinoma (27) etc. In colorectal cancer (CRC), glycosylation can impact cell migration, intercellular adhesion, actin polymerization, mitosis, cell membrane repair, apoptosis, cell differentiation, stem cell regulation, intestinal mucosal barrier integrity, immune system regulation, T cell polarization, and intestinal microbiota composition (28, 29). These functions are closely linked to the prognosis and development of CRC, including tumor occurrence, metastasis, immune regulation, and resistance to anti-tumor treatments (28–32). C1GALT1 (T-synthase), a key enzyme in the glycosylation process, has been identified as playing a role in colorectal carcinogenesis in recent years (33, 34). Given the well-established significance of glycosylation in cancer, this article aims to review the role of C1GALT1 in the complex glycosylation process and its relationship with colorectal cancer, providing evidence for the identification of specific therapeutic targets for CRC.

## 2 C1GALT1 in glycosylation

**Figure 2:**
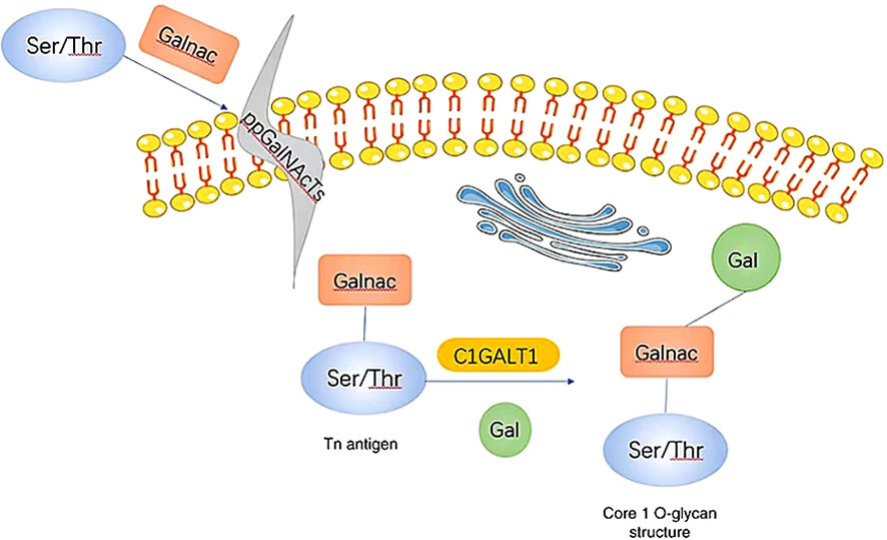
When GalNAc transferase is present, it facilitates the formation of a GalNAc α1-Ser/Thr structure (also referred to as the Tn antigen) with GalNAc on serine/threonine residues. Subsequently, C1GALT1, a core 1β1,3-galactosyltransferase, catalyzes the addition of Gal from UDP-Gal to the Tn antigen, resulting in the creation of a core 1 O-glycan structure. Gal, galactose; GalNAc, N-acetylgalactosamine; Tn, Thomsen-nouvelle; C1GALT1, core 1 β1,3-galactosyltransferase (33).

C1GALT1 (T-synthase) is a crucial mucin-type O-glycosyltransferase that functions as a type II transmembrane glycoprotein with endoplasmic reticulum (ER) and Golgi lumen-oriented catalytic domains (13, 35). As a glycosyltransferase, it plays a key role in the formation of the core 1 structure (Galβ1-3GalNAcα1-O-Ser/Thr) (33, 34). In most normal cells, C1GALT1 is essential for the immediate elongation and processing of GalNAc-type protein O-glycosylation (36). O-GalNAc glycans, also known as mucin O-glycans, are one of the most common post-translational modifications (18, 37). The sugars present in O-GalNAc glycans include GalNAc, Gal, GlcNAc, Fuc, and Sia, while Man, Glc, or Xyl residues are not expressed (37). The initial and fundamental step of O-GalNAc glycosylation involves the addition of α-linked GalNAc to Ser or Thr residues through a series of enzymes known as GalNAcTs3 or ppGalNAts or GALNT, resulting in the production of the Tn antigen within the Golgi apparatus. Subsequently, C1GALT1 catalyzes the formation of the core 1 structure (14, 33, 34, 37, 38). C1GALT1 is responsible for adding β 1-3 glycosidic bonds between Gal and GalNAc Ser/Thr residues to generate the T antigen (the core 1 structure) (25). The T antigen serves as a precursor for the subsequent extension and maturation of mucin-type O-glycans (24, 39). The Tn antigen consists of a single GalNAc residue connected to Ser/Thr α-O-Ser/Thr, serves as the foundation for the extension of O-glycans into various complex branching structures through consecutive glycosyltransferase reactions (**Figure 2**). The Tn antigen may continue to be catalyzed by ST6GalNAc, which will generate sialyl Tn antigen (sTn) (40, 41).However, the Tn antigen without sialic acid modification will be modified by C1GALT1 (core 1 synthase).The C1GALT1 modification process is to add a Gal to Tn antigen in order to form the T antigen (42). In normal tissues, Tn or T antigens continue to synthesize complex O-glycans with the catalysis of glycoenzymes. Consequently, the Tn antigen is uncommon in normal mucins but is often found at increased levels in tumor mucins (43), and its presence has been closely associated with poor prognosis and low overall survival rates (39).

C1GALT1 enzyme activity requires the presence and function of the molecular chaperone COSMC, which is located in the ER (44). In the ER, C1GALT1 is converted into its active and dimeric forms by the molecular chaperone COSMC before entering the Golgi matrix (45). Within the Golgi apparatus, C1GALT1 competes with two other types of glycosyltransferases (C3GnT and ST6GalNAC-I/II) to catalyze the addition of Gal to GalNAc α-Ser/Thr, initiating O-linked Mucin Glycan Formation and the Core-1 Structure (46).

**Figure 3:**
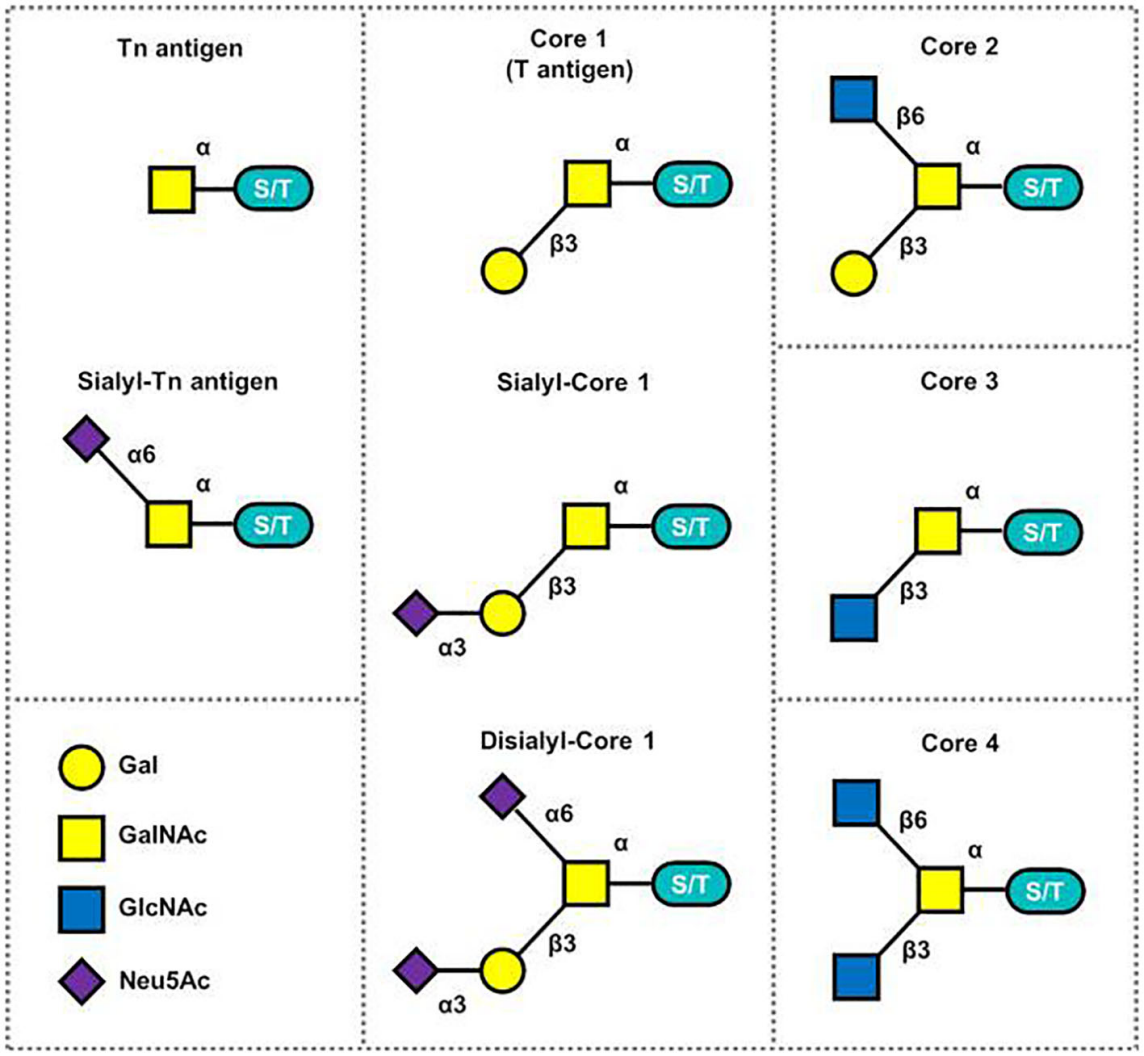
Mucin-type O-glycan core structures and key sialoglycoforms (51).

**Figure 4:**
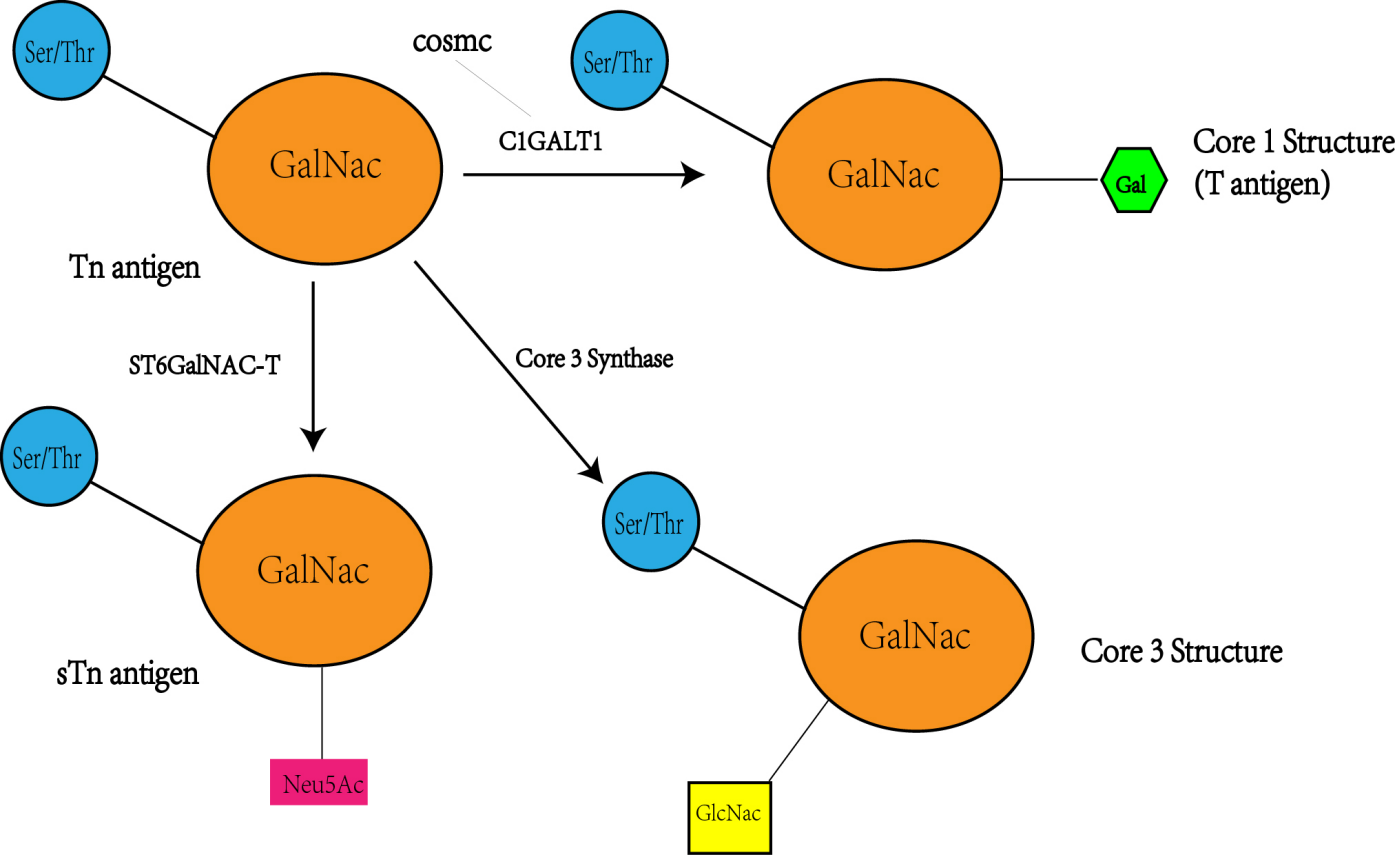
Tn antigen forms the T antigen i.e. core 1 structure under the action of C1GALT1, while the activity and level of CAGALT1 is regulated by COSMC. At the same time, Tn antigen can be converted to STn as well as core 3 structures catalyzed by the other two enzymes(ST6GalNAC; core 3 synthase i.e. β 3GnT6, C3GnT), so there may be a competitive relationship between these three enzymes, with a decrease in C1GALT1 levels accompanied by an increase in STn and core 3 structure levels.

The Tn antigen (GalNAcα 1-O-Ser/Thr) is an O-glycan commonly expressed in various types of human cancers, often resulting from incomplete glycosylation (47). It belongs to the category of tumor-associated carbohydrate antigens (TACA) found in human cancer. The most common TACAs formed from incomplete synthesis are GalNAcα-O-Ser/Thr (Tn, Thomsen Nouveau, CD175), Galβ1,3-GalNAcα-O-Ser/Thr (TF, Thomsen-Friedenreich, CD176, T antigen), Neu5Acα2,6-GalNAcα-O-Ser/Thr (sTn, sialyl Tn, CD175s) and Neu5Acα2,6- and Neu5Acα2,3-Galβ1,3-GalNAcα-O-Ser/Thr (2,6-sTF, 2,3-sTF) (47). In normal tissues, the Tn antigen is typically undetectable due to its efficient conversion into a broader range of glycans, primarily the core 1 structure (Galβ1-3GalNAcα-O-Ser/Thr, T or TF antigen) (43). The core 1 structure can further extend into the extended core 1 O-glycan (core 1 O-glycan), or branch into the core 2 structure or undergo sialylation (48). In the gastrointestinal tract (GI tract), the Tn antigen can be converted into the core 3 structure (34, 49), while in the normal colon, the primary O-glycan core structure is the core 3 structure (50). The core 4 structure is formed by adding Uridine Diphosphate (UDP) from the core 3 precursor to the core 3 infrastructure through C2GnT2-α-D-GlcNAcβ1,6-GlcNAc (51). As described above, Mucin-type O-glycan core structures and key sialoglycoforms such as the Tn antigen, the core 1 structure are shown in **Figure 3** and the process of structure formation is shown in **Figure 4**. However, incomplete glycosylation caused by various factors can lead to the production of TACAs as described above. These truncated O-glycans possess carcinogenic characteristics and can directly induce cell growth and invasion (52).

## 3 C1GALT1 in colorectal cancer

Based on the information provided, it seems that C1GALT1 plays a crucial role in the glycosylation process, affecting the production of Tn antigen and the subsequent formation of downstream proteins like the core 1 structure. The mechanism of action of C1GALT1 is multifaceted and involves the regulation of target protein expression, phosphorylation, and localization, ultimately controlling various biological processes such as tumor proliferation, migration, and adhesion (25, 53, 54). In colorectal cancer cells, elevated T-synthase activity and overexpression of Cosmc and T synthase have been observed (55, 56). Recent studies have shown that SP cells isolated from human umbilical cord mesenchymal stem cells (hUCMSCs) and human placental mesenchymal stem cells (hPMSC) altered the O-glycosylation status by increasing O-glycosyltransferase activity, thereby inhibiting the proliferation and migration of Tn CRC cells and promoting apoptosis of Tn CRC cells (57). Based on these findings and previous research on cancer, several hypotheses regarding the mechanism of C1GALT1 action in colorectal cancer (CRC) have been proposed.

### 3.1 C1GALT1 and TACA

To begin with, in conjunction with the aforementioned information, Tumor-Associated Carbohydrate Antigens (TACA) such as Tn and sTn are produced during the O-glycosylation process, and C1GALT1 plays a significant role in this process. Notably, the detection of Tn and sTn antigens is prevalent in samples of colorectal cancer, linking their presence to the likelihood of cancer spread and a negative outlook for the patient (20, 58, 59). This connection implies that the appearance of Tn and sTn within cells could be one of the initial steps in the development of colorectal cancer (34). Moreover, the neo- or over-expression of Tn, sTn, and T antigens is not limited to colorectal cancer but occurs in many types of cancer, including gastric, colon, breast, lung, esophageal, prostate, and endometrial cancer (60, 61). This widespread occurrence underscores the significance of these antigens in cancer biology, particularly in promoting tumor metastasis. Approximately 86% of primary and metastatic human CRC tissues express Tn epitopes (62). Research has shown that the Tn antigen can trigger the Epithelial-Mesenchymal Transition (EMT) via the H-Ras gene within the Ras/MAPK signaling pathway (56). Furthermore, this activation leads to a decrease in epithelial cell markers, such as snail, and increases the metastatic capabilities of colon cancer cells. Additionally, the targeted suppression of the C1galt1c1 gene using CRISPR/Cas9 technology results in increased levels of Tn antigen on the surface of the colorectal cancer cell line MC38 (MC38-Tnhigh) (63). In a study of pancreatic cancer, by using the CRISPR/Cas9 system to disrupt C1GALT1 in human pancreatic ductal adenocarcinomas cells, the results showed enhanced invasiveness and metastatic ability of these cells and increased production of Tn (64). These observations collectively suggest that C1GALT1’s regulation of Tn and sTn antigen production plays a significant role in the development and progression of colorectal cancer by influencing its ability to spread.

### 3.2 C1GALT1 and core 3 synthase

The core 3 structure is a kind of O-glycans which plays an important role in the differentiation of gastrointestinal goblet cells and the formation of mucosal barrier β Mucin type O-glycans synthesized by 1,3-N acetylglucosamine transferase 6 (core 3 synthase, β 3GnT6, C3GnT) (65). The core 3 structure is synthesized by β1,3-N-acetylglucosaminyltransferase 6 (B3GNT6 or core 3 synthetase), which adds GlcNAc with a β1,3-linkage to the Tn antigen (GalNAc alpha-serine/threonine), and since core 1 synthetase makes use of the same 3′-position of GalNAc found in the Tn antigen, the synthesis of core 3 may compete with core 1 synthesis (64). Normal colon mucosa predominantly express core 3 O-glycans (50). The absence of O-glycans compromises the colonic mucus barrier, leading to inflammation through the activation of caspase 1-dependent inflammasomes in colonic epithelial cells, a process mediated by the microbiota (65). Another research has shown a novel mechanism by which mucin-type core 3 O-glycan influences the epithelial-mesenchymal transition (EMT) and mesenchymal-epithelial transition (MET) plasticity of colorectal cancer (CRC) cells through a MUC1/p53/miR-200c-dependent signaling cascade (66). An experiment has demonstrated that the inhibition of C1GALT1 is accompanied by an increase in the expression of sialic acid Tn and GSL-II binding (core 3 structure) in human colorectal cancer cells (67). Thus overexpression of C1GALT1 may reduce the core 3 structure, thereby inducing colorectal cancer. Additionally, decreased expression of core 3 synthase is associated with lymph node and distant organ metastasis, leading to poor prognosis in CRC patients (66). In a mouse experiment that showed a significant increase in the incidence of colon-related diseases with increasing age, the expression of C1GalT1 increased 1-fold, whereas the expression of core 2 β1,6-N-acetylglucosaminyltransferase (C2GnT) and core 3 β1,3-N-acetylglucosaminyltransferase (C3GnT) declined 2- to 6-fold and 2-fold, respectively (68). Therefore, the overexpression of C1GALT1, leading to a reduction in the quantity or activity of core 3 synthase and subsequent decrease in the core 3 structure, may contribute to the development of colon cancer.

### 3.3 C1GALT1 and Cosmc

Despite its significance, the precise structure of C1GALT1 and the mechanisms of substrate recognition and catalysis remain elusive (69), underscoring a gap in our understanding of how these antigens are produced at the molecular level. A key aspect of this process involves the unique molecular chaperone of C1GALT1, known as Cosmc, which is essential for the proper folding of T-synthase in the endoplasmic reticulum. The dysregulation of glycosyltransferases like C1GALT1, molecular chaperones like Cosmc, or the cellular environment can lead to the abnormal regulation of O-glycans, contributing to the development and progression of various cancers. A study revealed that the upregulation of C1GALT1 was accompanied by an increase in Cosmc levels in colorectal cancer cells (66). Further experiments demonstrated that C1GALT1 was absent in Cosmc-deficient cancer cells, consistent with previous reports indicating that the presence of T-synthase relies on intact Cosmc (56). It has also been observed that T-synthase activity and Cosmc, a crucial chaperone for its expression, were lower in Tn-positive CRC tissues compared to negative tissues (62). Others have proposed that the characteristic truncation of O-glycans found in pancreatic cancer and most epithelial cancers is not due to somatic mutations, but at least partially due to epigenetic silencing of COSMC companion genes caused by promoter hypermethylation (52). While, Sun (34) et al. have found that LOH that is a common mechanism of loss of gene function in tumorigenesis occurs in Cosmc, but not C1GALT1, through studies of CRC cells. The expression and mutation of Cosmc significantly impact C1GALT1 activity, levels, and Tn antigen expression, thereby playing a crucial role in the development of colorectal cancer.

### 3.4 Downstream regulators of C1GALT1

C1GALT1, a key player in colon cancer pathogenesis, significantly influences the behavior and properties of cancer cells. This enzyme’s overexpression alters O-glycans on Fibroblast Growth Factor Receptor 2 (FGFR2), a receptor tyrosine kinase overexpressed in colorectal cancer (53). FGFR2 is crucial for cellular processes like proliferation, survival, migration, and differentiation (70, 71). The modification of FGFR2 by C1GALT1, as evidenced by the presence of sTn on FGFR2, enhances its phosphorylation, promoting invasive behavior and cancer stem-like properties in colon cancer cells (53, 72–76). Furthermore, C1GALT1’s role extends to the epithelial-mesenchymal transition (EMT) in cancer cells. A deficiency in C1GALT1 leads to the classical EMT profile, characterized by a reduction in E-cadherin (an epithelial marker) and an increase in mesenchymal markers like snail and fibronectin. This change in cellular markers indicates a transformation in the cancer cells’ behavior and properties (77). Additionally, C1GALT1 suppression impacts tumor cell interactions and activities. It notably reduces galectin-3-mediated tumor cell-cell interaction and the promotion of tumor cell activities by galectin-3 (69). A study on colon cancer cells found that the expression of silyl-Tn was associated with an increase in the α2,6-carbamoyltransferase gene (ST6GALNAC1) and a decrease in the core 1 synthase gene (C1GALT1) in LS174T cells, by qRT-PCR (78). A recent study on endometrial cancer showed that low expression of C1GALT1 induced overexpression of ANXA1 in ECC-1 cells, which were characterized by higher proliferation, invasion, migration, colony formation and angiogenesis (79). Besides, C1GALT1 is able to modify O-linked glycosylation on integrin α5, thereby modulating activation of the PI3K/AKT pathway in gastric cancer cells (80). Also in pancreatic cancer, C1GALT1 knockdown significantly inhibited cell adhesion to the extracellular matrix (ECM), which was associated with a decrease in FAK phosphorylation at Y397/Y925 as well as changes in O-glycans on integrins (including β1, αv and α5 subunits) (81). In addition, C1GALT1 affects the migratory ability, proliferation and colony formation of bladder cancer cells through a mechanism of miR-1-3p/cHP1BP3 axis deregulation and shows tumor suppressor activity in bladder cancer cells (82). Recent research has unveiled that knockout of the Zn2+-transporter SLC39A9 (ZIP9), alongside the well-described targets C1GALT1 (C1GalT1) and its molecular chaperone, C1GALT1C1 (COSMC), results in surface-expression of cancer-associated O-glycans (83). In gastric cancer, C1GALT1 promotes EPHA2 phosphorylation and enhances soluble Ephrin A1-mediated migration mainly by modifying the O-glycosylation of EPHA2, thereby affecting the cell invasiveness of gastric cancer cells (84). This further underscores C1GALT1’s significant role in the progression and characteristics of epithelial cancers, including colon cancer.

### 3.5 C1GALT1 and MUC1

Due to the high concentration of O-linked GalNAc on mucin proteins, further refinement produces what is commonly referred to as mucin-type O-glycans. The exposed Tn antigen is the ligand of Ca2^+^dependent C-type lectin receptor MGL (macrophage galactose type lectin/CD301/CLEC10A) (85), MGL specifically recognizes tumor derived mucin MUC1 by binding to Tn antigen (86). Mucin 1 (MUC1) is a single channel type I transmembrane protein with a highly glycosylated extracellular domain, which is usually located at the root tip edge of epithelial cells and plays a protective role in lower epithelial cells (87). Altered glycosylation of the oncoprotein MUC1 commonly occurs in chronic inflammation, including ulcerative colitis, and this aberrantly glycosylated MUC1 promotes cancer development and progression (88). While, MUC1 deficiency has been shown to suppress inflammation, inhibit tumor progression, increase the abundance of CD8 T-lymphocytes, and decrease the abundance of macrophages in colon tumors (89). In various epithelial cancers MUC1 has abnormal glycosylation and overexpression (90, 91). Increased expression of MUC1 in CRC is associated with worse prognosis metastasis (87, 92). Besides, MUC1 participates in complex immune process and has immunomodulatory effect (93, 94).And, the latest research has found that crosstalk between macrophages and colonocytes increasing MUC1-sTn expression (88). The expression of T, sTn on tumor mucins such as MUC1 which plays as various selectins of ligands, caused by abnormal glycosylation promotes metastasis and spread (95). Mucins are carriers of selectin ligands, facilitate metastasis and spread by forming aggregates with cells which express selectins on their surface (96–98). Cancer cells use this ability of mucin to escape immune surveillance (95). Therefore, mucins play an important role in cancer metastasis and diffusion.

The N-terminal of MUC1 contains VNTR fragment, which contains serine and threonine residues and provides a number of O-glycosylation sites (99), including T and sTn antigens (100). In MUC1 with insufficient glycosylation, the VNTR region containing exposed cryptic peptide epitopes has been proved to elicit strong humoral- and cell-mediated immune responses (95). Based on this theory, in recent years, many studies have found the target of MUC1 anti-tumor vaccine, and the results showed that the survival rate was improved (101) and the metastatic foci were reduced in the mouse model. In the experiment of human colorectal cancer cells, anti MUC1 shows potential for colorectal cancer treatment (102, 103). Targeting MUC1-C to inhibit the AKT-S6K1-elF4A pathway regulates TIGAR translation in colorectal cancer and inhibits the growth of colon cancer cells *in vitro* (104). Glycosylation of MUC1 depends on the biomolecules it recognizes, such as, for example, GalNAc transferase, visfatin lectin, antibodies and other glycosyltransferases (105), while a research found that anti MUC1 treatment can inhibit C1GALT1 at protein level (106). In other words, the impact of C1GALT1 on MUC1 glycosylation has a certain influence on the development of colorectal cancer, although the specific mechanism is not yet fully understood. It has been found in the study of esophageal cancer, another epithelial cell carcinoma that the expression level of C1GALT1 was positively correlated with MUC1 O- glycosylation, and the co expression of MUC1 and C1GALT1 was negatively correlated with survival rate (26). Therefore, it is plausible to hypothesize that a similar pathway involving MUC1 may be involved in the development of colorectal cancer. However, further studies are still required to elucidate the exact mechanisms underlying this relationship.

## 4 Summary

**Figure 5:**
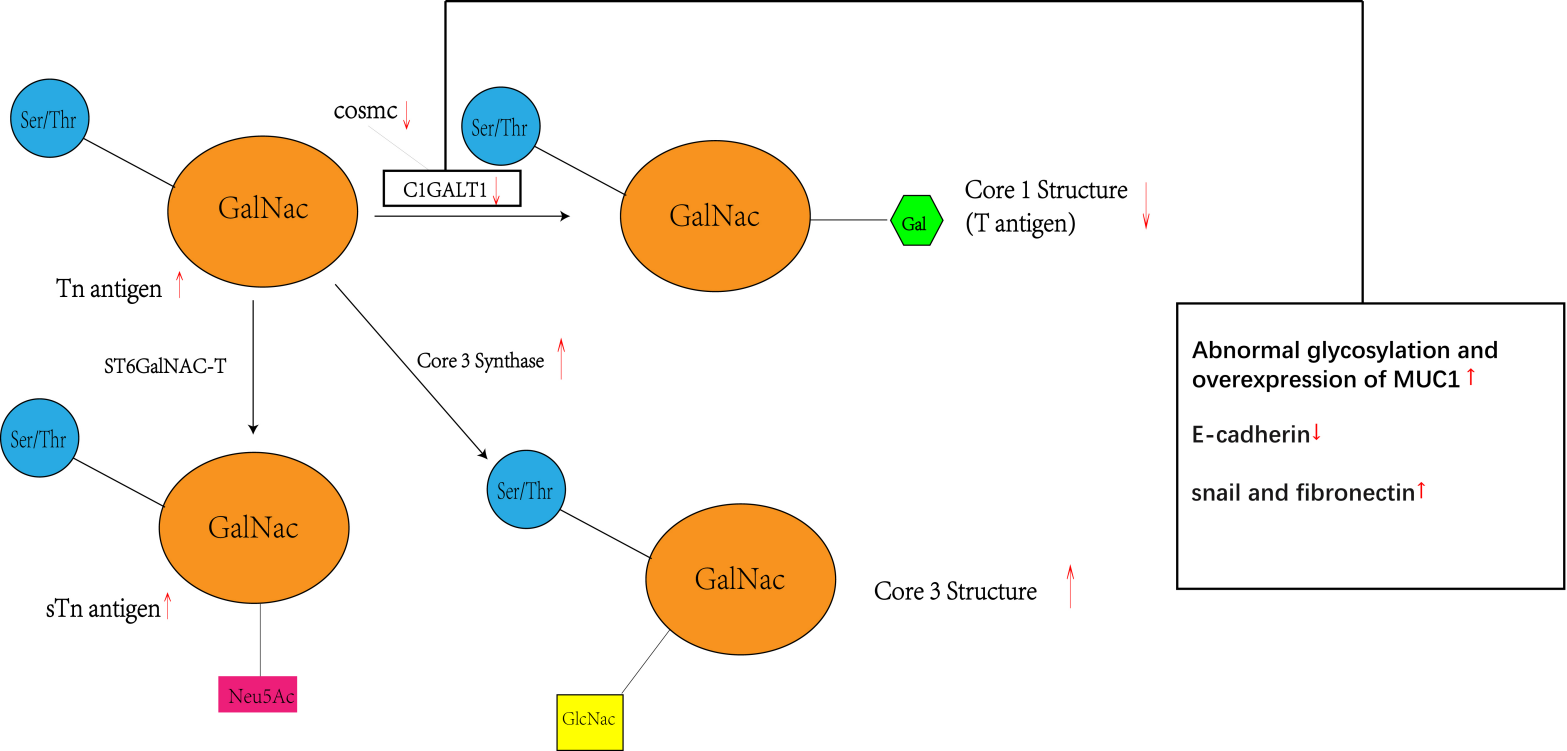
Inhibition of C1GALT1 leads to elevated Tn antigen, and due to competition, inhibition of C1GALT1 leads to a relative dominance of ST6GalNAC and core 3 synthase, resulting in elevated levels of sTn antigen and core 3 structure and decreased levels of core 1 structure. At the same time C1GALT1 deficiency directly induces the classical EMT signature of cancer cells, i.e., a marked decrease in the typical epithelial cell marker E-cadherin and enhanced expression of isochronous stromal markers, including snail and fibronectin. Meanwhile C1GALT1 inhibition induces abnormal glycosylation and overexpression of MUC1.

The prospect of C1GALT1 in colorectal cancer (CRC) is multifaceted, mainly in terms of its key role in the process of tumorigenesis and progression as well as its potential as a potential therapeutic target. C1GALT1 produces tumor-associated carbohydrate antigens (TACA), such as Tn and sTn antigens, through the process of O-glycosylation, and these antigens have been associated with the spread of colorectal cancer and poor patient prognosis. The O-glycosylation of the core 3 structure is essential for the differentiation of gastrointestinal tract cup cells and the formation of the mucosal barrier.Regulation of core 3 structure expression by C1GALT1 may affect the development of colorectal cancer, and its overexpression may reduce the core 3 structure, which can induce cancer. Cosmc acts as a molecular chaperone for C1GALT1 and is essential for the correct folding of T-synthase in the endoplasmic reticulum.Expression and mutation of Cosmc significantly affects the activity of C1GALT1 and the expression of Tn antigens, emphasizing its role in CRC. C1GALT1 affects the behavior of cancer cells by altering the O-glycosylation on FGFR2, which promotes cancer cell invasiveness and stem cell-like properties.C1GALT1 also affects the epithelial-mesenchymal transition (EMT) of cancer cells, indicating its important role in cancer progression. Aberrant glycosylation of MUC1 plays a role in cancer development and progression.The effect of C1GALT1 on MUC1 glycosylation plays a role in CRC development, and cancer therapies targeting MUC1 show potential, suggesting a potential effect of C1GALT1 on MUC1 glycosylation. The specific interactions and regulatory mechanisms between C1GALT1 and TACA, core 3 synthase, Cosmc, downstream regulators, and MUC1 were further explored to better understand its role in CRC. Based on the key role of C1GALT1 in CRC, the development of inhibitors or activators targeting its activity, expression or its interaction with other molecules as a new therapeutic strategy. Considering the interactions of C1GALT1 with multiple molecules and pathways, exploring combinatorial therapeutic strategies that combine treatments targeting C1GALT1 with other therapeutic approaches (e.g., chemotherapy, immunotherapy) to improve therapeutic efficacy. The pathway described above is specifically shown in **Figure 5**. This review highlights the potential mechanisms by which C1GALT1 contributes to the carcinogenesis of CRC. It holds promise as a specific therapeutic target for CRC. Furthermore, the precise mechanism of C1GALT1 in CRC has yet to be fully elucidated, presenting strong prospects for further research. The next step could be to develop inhibitors targeting C1GALT1 activity or expression, and assess their effects on CRC cell proliferation and invasion through in vitro and in vivo experiments. Also explore the molecules and pathways that interact with C1GALT1 (including COSMC and Core 3 Synthase, etc.) and develop corresponding activators or inhibitors to modulate its role in CRC. This may be a promising research direction.

## Author contributions

HT: Writing – review & editing, Supervision, Funding acquisition, Conceptualization. J-L Yu: Data curation, Writing – original draft. XC: Writing – review & editing, Data curation. QG: Writing – review & editing, Formal analysis. JL: Writing – review & editing, Formal analysis. YL: Writing – review & editing, Resources.
